# A novel chitosan agarose nanocopper composite film (Cs/Agr/Cu-Cu_2_O NPs) using *Aspergillus niger* K Y 401431: preparation, characterization and evaluation of their antibacterial activity

**DOI:** 10.1007/s13205-025-04471-7

**Published:** 2025-08-20

**Authors:** Heba A. Abd El Rahman, Amany A. El-Sharif, Ahmed M. Youssef, Sanaa K. Gomaa, Sayeda S. Mohamed, Heba A. El Refai

**Affiliations:** 1 Chemistry of Natural and Microbial Products Department, Pharmaceutical and Drug Industries Institute, National Research Center, Dokki, Giza, Egypt; 2 Department of Microbiology and Immunology, Faculty of Pharmacy, Al-Azhar University, Cairo, Egypt; 3 Packaging Materials Department, National Research Centre, 33 El Bohouth St. (Former El Tahrir St.), Dokki, P.O. 12622, Giza, Egypt

**Keywords:** Cu/Cu_2_O NPs, *Aspergillus niger* K Y 401431, Cs/Agr/Cu-Cu_2_O bionanocomposite film, Characterization, Antibacterial activity

## Abstract

*Aspergillus niger* K Y 401431 was used in the present study for biosynthesis of copper/copper oxide nanoparticles. *A niger* K Y 401431 was checked for the production of the carcinogenic mycotoxin ‘ochratoxin A’ to prove the safety of the strain and the culture filtrate was free from ochratoxin A. For the optimized production of Cu/Cu_2_O NPs, the influence of some parameters was investigated. 10 mM of CuSO_4_ was optimal for nanoparticle production, well-defined Cu/ Cu_2_O NPs formation occurred after 240 min of incubation using culture filtrate of *A. niger* K Y 401431developed under submerged culture conditions for 3 days culture age. The biosynthesized copper nanoparticles were characterized by UV–visible spectroscopy, transmission electron microscopy (TEM), energy dispersive X-ray (EDX), Fourier-transform infrared spectroscopy (FT-IR), dynamic light scattering (DLS), X-ray diffraction (XRD). The characterization of Cu/Cu_2_O NPs revealed the formation of crystalline spherical shaped (Cu/Cu_2_O NPs) with 4–10 nm size. The cytotoxic efficacy was investigated against three cancer cell lines MCF7 (Human Caucasian breast adenocarcinoma), HEPG2 (human hepatocellular carcinoma cell line) and A549 (Lung carcinoma cell line) using MTT (3-(4, 5-dimethylthiazol-2-yl)-2, 5-diphenyltetrazolium bromide) assay. The cytotoxic effect of the biosynthesized Cu/ Cu_2_O NPs on A549, HEPG-2, and MCF-7 showed IC_50_ of 20.3, 56.9, and 43.3 µg / ml, respectively. In this study, the biosynthesized Cu/ Cu_2_O NPs were used for the preparation of chitosan/agarose/copper bionanocomposite film by casting method. The Cs/Agr/ Cu-Cu_2_O bionanocomposite films were investigated using SEM, XRD, and FT-IR. The prepared bionanocomposite film displayed enhanced antibacterial activity against pathogenic bacteria.

## Introduction

Antimicrobial resistance (AMR) increased lately due to the overuse of the available antimicrobial medicines, so several approaches have been concerned with the development of new and improved antimicrobial agents against many resistant pathogens that represents a serious global hazard threat to health (Ren et al. 2009; Blecher et al. 2011; Farokhzad and Langer 2009; Kaweeteerawat et al. 2017; Pelgrift and Friedman 2013; Zhang et al. 2008; Flores-Rábago et al. 2023). Nanomaterials offer a promising trend to treat multidrug resistant bacterial infections. Nanotechnology is a significant area of study that deals with the design and manipulation of small particles, or nanoparticles, that are 100 nm or smaller. These particles have a variety of properties that set them distinguished from their bulk material, including electrical, optical, magnetic, catalytic, and biological activity, as well as a broad range of anti-microbial activity against various species of gram-positive and gram-negative bacteria and fungi (Dastjerdi et al. 2010; Chandrakala et al. 2022; Vaishnavi et al. 2020; Joy et al. 2023; Ozdal and Gurkok 2022; Maheswari et al. 2023).

Global emphasis has been placed on the development of easy, affordable, and efficient techniques for the synthesis of nanomaterials. Microbial-based biosynthesis approaches have become a clean, sustainable, and suitable alternative for chemical and physical techniques (Shantkriti and Rani 2014). The production of nanoparticles involves a variety of bio-resources, such as bacteria, viruses, plants, algae, and yeast (Malik et al. 2014). Inorganic nanoparticles can be produced intracellularly or extracellularly (Vetchinkina et al. 2018; Murillo-Rábago et al. 2022**)**. Fungi are favored over bacteria because of their exceeding metal tolerance and bioaccumulation potential (Sastry et al. 2003). In addition, ease of scaling up and biomass handling and secretion of large amounts of enzymes make fungi more feasible than bacteria (Sheikhloo et al. 2011; Noor et al. 2020).

*Aspergillus niger* is a filamentous fungus commonly found in soil producing a variety of enzymes such as oxidative and hydrolytic enzymes and can absorb metal ions from aqueous solution (Ingle et al. 2008; Xie and Kang 2009). It is one of the most important industrial filamentous fungal species used in biotechnology (Pel et al. 2007; Andersen et al. 2011) and is extensively used for production of extracellular enzymes (Bellaouchi et al. 2021) and organic acids such as citric acid (Show et al. 2015). The US Food and Drug Administration has classified *A. niger* as GRAS (Generally Regarded as Safe) for certain industrial production processes (Blumenthal 2004).

The detection of *A. niger* strains producing ochratoxin A (OTA) raised concerns for their biotechnological safety (Abarca et al. 1994). According to the IARC (1993), OTA is a strong nephrotoxic possessing immunosuppressive, carcinogenic and teratogenic characteristics. There is an opportunity that some industrial strains of *A. niger* could produce ochratoxins, since up to 41% of all *A. niger* isolates were reported positive for ochratoxin production (Frisvad et al. 2011). Hence, it's critical to check strains of every Aspergillus species for the production of mycotoxin.

Metal nanoparticles and metal oxide nanoparticles (MONPs) have received significant attention as antibacterial agents because of their high surface area to volume ratio, which makes nanoparticles effective at low concentrations (Sundaresan et al. 2012). Copper nanoparticle synthesis specifically has attracted more interest compared to other NPs because of their useful properties achievable at much less cost than silver and gold (Han et al. 2006).

Copper nanoparticles have been reported to have antibacterial properties (Cioffi et al. 2005). Copper nanoparticles are capable of destroying and disrupting microbial cell components through redox reactions. Therefore, their antibacterial properties have been utilized to enhance some polymers utilized in food packaging (Youssef et al. 2020; Zhang et al. 2023; Adeyemi and Fawole 2023). Also, copper is a potential material for electronic systems because of its electrical and thermal conductivity (Kim et al. 2009) and conductive inks (Lee et al. 2008).

In recent years, the worldwide interest to develop bioactive nanocomposites increased (Song et al. 2018; Youssef & El-Sayed 2018). The potential for customized materials in a variety of industries has been greatly increased by recent developments in nanoparticle synthesis. Megalibraries, spherical nucleic acids, high-entropy alloys, and sophisticated laser and microwave techniques provide previously unheard-of control over the size, composition, and functioning of nanoparticles. These developments underscore the increasing significance of nanoparticles in contemporary science and industry by opening the door for the creation of more effective catalysts, energy materials, and biological applications (Youssef & El-Sayed 2018**)**.

Natural polymers, such as polysaccharides or proteins can be used to construct the bulk material component of nanocomposites. Chitosan (Cs); a linear cationic polysaccharide found in nature; has gained momentum because of its bioactivity with highly functional amounts of amine and hydroxyl groups (Jayakumar et al. 2010). They are widely recognized for having a broad range of applications and are frequently suggested for use in the food, pharmaceutical, and biomedical industries. Chitosan is being considered as a stabilizer for the CuNPs because of its capacity to chelate metals, suggesting it as an ideal candidate for biocomposite formation (Hardy et al. 2004). Referring to their availability, biocompatibility, and lower toxicity than dangerous chemical solvents; biopolymers are increasingly being used as stabilizers for the synthesis of CuNPs (Usman et al. 2012). It is important to note that chitosan's film-forming qualities and wide range of antibacterial and antifungal properties make it a viable material for packaging films. The antibacterial activity of the Cu-chitosan nanoparticle composite was found to be higher than that of the copper nanoparticles and chitosan alone, indicating that the inclusion of chitosan stabilizer significantly improves the antimicrobial activity of CuO NPs (Syam et al. 2017). The chitosan/carboxymethyl cellulose/zinc oxide bionanocomposite film was reported to enhance the Egyptian soft white cheese shelf life (Youssef et al. 2016).

Agarose (AG) is a biocompatible linear polysaccharide that may be derived from marine red algae. Agarose has the unique capacity to create a thermally reversible gel because of its particularly hydrophilic and macroporous structure. By altering its composition, agarose's mechanical characteristics can be readily adjusted and are comparable to those of tissues. Agarose has been seen as a promising candidate for use in biomaterials because of its great gelling power, biodegradability, and renewability (Deszczynski et al. 2003; Lahooti and Sefton 2000; Watase et al. 1989). For the delivery of berbamine, chitosan–agarose composite microspheres were successfully developed. It was discovered that incorporating agarose to chitosan microspheres enhanced the drug adsorption and release capabilities (Hu et al. 2012). Hu et al. (2016) reported that chitosan–agarose composite films show immense potential for application in biomedical materials and food packaging.

Here in the current study, we report a simple green approach for biosynthesis of copper/copper oxide nanoparticles by the extracellular culture filtrate of *A. niger* KY401431 with chitosan agarose as stabilizer. The bio-synthesized copper nanoparticles and their chitosan-agarose nanocomposite were characterized and their antimicrobial properties were investigated. Furthermore, evaluation of the OTA production by the *A. niger* KY401431 strain used in this study was inspected. This new Cs/Agr/Cu- Cu_2_O bionanocomposite film can be employed as a sustainable and environmentally friendly food packaging material.

## Materials and methods

### Materials

Copper sulphate, Ochratoxin A (OTA) standard, Glacial acetic acid (HAc) used as the solvent for chitosan and all other reagents/chemicals were of analytical grade and were purchased from Sigma Aldrich (St. Louis, MO, USA). All solvents employed in HPLC analysis were HPLC grade. Chitosan (CH) powder (medium molecular weight MW = 161,000 142 g/mol, degree of deacetylation, DD = 75.6%), and viscosity 1406 m.Pas. All utilized media components were of highest purity and were obtained from Oxoid (UK), Difco (USA), and Sigma–Aldrich (St. Louis, MO, USA).

### Methods

#### Screening and maintenance of fungal strains for Cu/Cu_2_O NPs

In the present study, seven fungal strains (*Penicillium chrysogenum*, *Aspergillus ochraceus*, *Aspergillus niger* KY401431, *Rhizopus oryzae*, fungal isolate 1, fungal isolate 2, fungal isolate 3) were used for copper/copper oxide nanoparticles production. All pure cultures were maintained on Potato Dextrose Agar medium (PDA) slants at 4 ℃.

#### Extracellular filtrate preparation

The tested cultures were grown for 5 days on PDA slants. 10 ml inoculum of each culture was transferred into 100 ml sterile Sabouraud Dextrose broth. Cultures were incubated for 72 h, at 30 ℃ and 150 rpm in an incubated arbitrary shaker. The control experiments were carried out with un-inoculated media and with CuSO_4_ solution. Biomass of each tested culture was filtered with Whatman filter paper (No. 1). The obtained culture filtrates were then used as reducing agents for the biosynthesis of Cu/Cu_2_O NPs.

### Biosynthesis of Cu/Cu_2_O NPs

Biosynthesis of Cu/Cu_2_O NPs was done according to Nassar et al. (2023), with modification. The obtained culture filtrate was added dropwise with stirring to the reaction vessel containing 10 mM copper sulphate dissolved in 2 ml deionized water. The reaction solution was heated to 80 ℃. 0.1 M NaOH was added drop by drop under continuous stirring until a red precipitate is formed. The reaction was allowed to settle overnight. The supernatant was discarded carefully and the precipitate (Cu/Cu_2_O NPs) was washed using deionized water centrifuged at 5000 rpm for 10 min and washing with deionized water was repeated three times through consecutive precipitation and re-suspension in deionized water, and then washing with absolute ethanol. The precipitate was dried at 60̊ C and kept for further studies.

### Detection of ochratoxin a production by *Aspergillus niger* KY401431

#### Extraction of the toxins from the culture media

*Aspergillus niger* KY401431 cultures were grown on yeast extract sucrose (YES) agar medium (Fisher, Loughborough, Leicestershire, UK) for 10 days at 25 ℃ until sporulation occurred. A total of five agar plugs were randomly collected using a sterile cork borer and transferred into 2 ml vials. For ochratoxin A (OTA) extraction from the agar plug, 750 μl methanol was added, the sample shaken for 30 min and centrifuged for 10 min at 15,000 g. After filtration of the supernatant (nylon syringe filter, 0.22 µm pore size, Fisher), the samples were analysed by HPLC. Samples were taken as positive for the toxin presence when a peak was obtained at a retention time similar to the standard (Sultan and Magan 2010).

#### High performance liquid chromatography (HPLC) analysis

The HPLC system used for AF and OTA analyses was an Agilent 1200 series system (Agilent, Berks., UK) with a fluorescence detector (FLD G1321A), an auto sampler ALS G1329A, FC/ALS therm G1330B, Degasser G1379B, Bin Bump G1312A and a C18 (Phenomonex, Luna 5 micron, 150 × 4.6 mm) column joined to a pre-column (security guard, 4 × 3 mm cartridge, Phenomenex Luna). The mobile phase was acetonitrile (57%)/water (41%)/acetic acid (2%) was isocratically used at flow rate of 1 ml min^−1^ at 333 nm excitation, 460 nm emission wavelengths. The run time for samples was 15 min.

### Optimization of Cu/Cu_2_O NPs biosynthesis conditions

Different concentrations (2.5, 5, 10, 20 and 30 m M) of CuSO_4_ solution were analyzed. The culture filtrate utilized for Cu/Cu_2_O NPs biosynthesis was obtained from submerged culture as mentioned in the previous Sect. (2.2.1) and a parallel solid-state culture of *Aspergillus niger* was conducted under static conditions to compare the effect of submerged fermentation (SmF) versus solid state fermentation (SSF). The effect of culture age (3–14 days) on biosynthesis of Cu/Cu_2_O NPs was also studied. Nanoparticles synthesis was recorded at different time intervals (30–240 min) to study the effect of reaction time on nanoparticle production. The absorbance of the reaction mixture for each treatment was measured spectrophotometrically.

### Characterization of biosynthesized Cu/Cu_2_O NPs

The Cu/Cu_2_O NPs were characterized using UV–Visible spectrophotometer (jascoV-730) to determine their surface plasmon resonance peaks. The morphology and size and the selected area diffraction pattern (SAED) were investigated using high resolution transmission electron microscope HRTEM (JEOL JEM-1200, Japan). SEM conjugated energy dispersive X-ray spectroscopy (EDX) using Field emission scanning electron microscope (FESEM) model Quanta FEG 250 was used to investigate the elemental composition of the biosynthesized nanoparticles. Attenuated total reflection (ATR) FTIR spectrum was acquired using a Vertex 80 FTIR spectrometer equipped with Platinum Diamond ATR. The average hydrodynamic diameter and the size distribution of sample were measured using a particle size analyzer (Nano-ZS, Malvern Instruments Ltd., UK). The X-ray diffraction (XRD) pattern was measured by the modern diffractometer Bruker d8 advance, using copper source at 40 Ma, 40 kV, in the 2ϴ range 5–80̊ and step 0.05̊.

### Chitosan/agarose/copper–copper oxide (Cs/Agr/Cu-Cu_2_O) bionanocomposite film preparation

The films were fabricated by the casting method (Hu et al. 2016; Youssef et al. 2016). The Cs/Agr/Cu-Cu_2_O bionanocomposites were prepared by the following procedure, Chitosan was mixed with acetic acid solution (2 wt% in water) at 60 °C in order to form a homogenous viscous solution (1 wt%). Separately, Agarose aqueous solution was achieved by dissolving agarose powder in hot distilled water at 100 °C stirring for 0.5 h (1 wt%). Cs/Agr blends were obtained by mixing the two polymer solutions (50:50 v/v). Then, the composites were prepared by adding the proper amount of Cu-Cu_2_O NPs to reach different ratios (1, 5, 10 wt %). They were prepared by steadily adding the Cu-Cu_2_O NPs suspension (1 mg/mL in water) to the Cs/Agr solution and sonicating for 2 h at 25 ℃ (Q500 Sonicator; sonication power, 500 Watts, frequency, 20 kHz and amplitude 50%). The homogeneous CS/Agr/Cu-Cu_2_O NPs suspensions were poured onto flat silicon-coated Petri dishes (60 mm × 15 mm) and left at room temperature for 72 h to evaporate the solvent. After that the dried films were peeled off from the mold using a spatula and stored in the desiccator prior to characterization.

### Characterization of Cs/Agr/Cu-Cu_2_O bionanocomposite film

The structural features of the Cs/Agr/Cu-Cu_2_O bionanocomposite films were performed using a Vertex 80 FTIR spectrometer from Bruker Optik GmbH, Germany, equipped with Platinum Diamond ATR in the spectral range of 4000–400 cm^−1^ with the resolution of 4 cm^−1^ to measure Attenuated total reflection (ATR) FTIR spectra. Because ATR-FTIR can examine the chemical structure and functional groups of the Cu/Cu2O nanoparticle-based bionanocomposite films, it was selected as a characterisation approach. It is ideal for thin-film analysis, involves little sample preparation, and is non-destructive. ATR-FTIR gave important information about the stability and composition of the film by assisting in the identification of the functional groups of the constituents (chitosan, agarose, and Cu/Cu2O) as well as any interactions between them.

The morphology of the CS/Agr/Cu-Cu_2_O bionanocomposite was examined by SEM (Quanta model FEG 250; Thermo Fisher Scientific, Japan). X-ray diffraction studies were performed with an X-ray diffractometer (Bruker d8 advance, Germany) with Cu Kα1 radiation to determine the structure of a sample. The X-ray source was operated at 40 kV and 40 m A, in the 2ϴ range 5̊−80̊ and step 0.05̊.

### Biological activities

#### [2, 2-Diphenyl-1-picrylhydrazil (DPPH) radical scavenging assay] of biosynthesized Cu/Cu_2_O NPs

The scavenging activity for DPPH free radical was measured according to Zhao et al. (2006) with some modifications. Samples (100 µl) were mixed with 900 µl of 0.1 mM DPPH solution in methanol. The mixture was shaken vigorously and allowed to reach a steady state at temperature 37 ℃. Decolourization of DPPH was determined by measuring the absorbance at 517 nm, and the DPPH radical scavenging was calculated according to the following equation:

% scavenging activity = (A_1_-A_2_/A_1_) × 100.

Where A_1_ was the absorbance of the DPPH solution without extract and A_2_ was the absorbance of DPPH with the extract. Ascorbic acid was taken as the standard. All the tests were performed in triplicate.

#### In vitro cytotoxicity

The cytotoxic efficacy of green synthesized Cu/Cu_2_O NPs was investigated against three cancer cell lines MCF7 (Human Caucasian breast adenocarcinoma), HEPG2 (human hepatocellular carcinoma cell line) and A549 (Lung carcinoma cell line). The three cell lines were obtained from Bioassay-Cell Culture Laboratory, National Research Centre, Dokki, Cairo 12,622, Egypt. Cell viability was assessed by the mitochondrial dependent reduction of yellow MTT (3-(4, 5-dimethylthiazol-2-yl)−2, 5-diphenyl tetrazolium bromide) to purple formazan (Mosmann 1983).

Procedure: All the following procedures were done in a sterile area using a Laminar flow cabinet biosafety class II level (Baker, SG403INT, Sanford, ME, USA). Cancer Cells individually (HEPG2, A549 and MCF7) were suspended in DMEM-F12 medium, 1% antibiotic–antimycotic mixture (10,000U/ml Potassium Penicillin, 10,000 µg/ml Streptomycin Sulfate and 25 µg/ml Amphotericin B) and 1% L-glutamine at 37 ºC under 5% CO2.

Cells were batch cultured for 10 days, then seeded at concentration of 10 × 10^3^ cells/well in fresh complete growth medium in 96-well microtiter plastic plates at 37 ºC for 24 h under 5% CO2 using a water jacketed Carbon dioxide incubator (Sheldon, TC2323, Cornelius, OR, USA). Media was aspirated, fresh medium (without serum) was added and cells were incubated either alone (negative control) or with different concentrations of sample to give a final concentration of (100–50-25–12.5–6.25–3.125–1.56 and 0.78 ug/ml). After 48 h of incubation, medium was aspirated, 40ul MTT salt (2.5 μg/ml) were added to each well and incubated for further four hours at 37 ℃ under 5% CO2. To stop the reaction and dissolving the formed crystals, 200 μL of 10% Sodium dodecyl sulphate (SDS) in deionized water was added to each well and incubated overnight at 37 ℃. A positive control which composed of 100 µg/ml was used as a known cytotoxic natural agent who gives 100% lethality under the same conditions (Thabrew et al. 1997; El- Menshawi et al. 2010; El-Baz et al. 2018).

The absorbance was then measured using a microplate multi-well reader (Bio-Rad Laboratories Inc., model 3350, Hercules, California, USA) at 595 nm and a reference wavelength of 620 nm. A statistical significance was tested between samples and negative control (cells with vehicle) using independent t-test by SPSS 11 program. DMSO is the vehicle used for dissolution of plant extracts and its final concentration on the cells was less than 0.2%. The percentage of change in viability was calculated according to the formula:

((Reading of sample/Reading of negative control) −1) × 100. Anesthetic procedures and handling with cancer cell lines complied with the ethical guidelines of the Medical Research Ethical Committee of the National Research Centre in Egypt (Approval no: 01241124).

#### Antibacterial activity of Cs/Agr/Cu-Cu_2_O bionanocomposite film

Qualitative evaluations were carried out according to (Sultan et al. 2022**).** The inoculation of pathogenic microorganisms used in this study were Gram-positive bacteria [*Bacillus cereus ATCC 6629, Staphylococcus aureus ATCC 6538 & Staphylococcus epidermidis ATCC 12228]*and Gram-negative bacteria [*Escherichia coli* ATCC 25922 *& Pseudomonas aeruginosa* ATCC27853] was prepared from fresh overnight broth cultures using nutrient broth medium that were incubated at 37 °C.

The inoculum size of each pathogenic strain was prepared and adjusted to approximately 0.5 McFarland standard (1.5 × 10^8^ CFU/ml) (McFarland 1907**)**, 25.0 µL inoculum size of each microorganism strain was separately inoculated into each plate containing 50.0 mL of the sterile nutrient agar medium (NA).

After the inoculated media cooled and solidified, 50 µl of Cu/Cu_2_O NPs suspension and sterile small equivalent specimens of the films [(Cs/Agr film), (1% Cs/Agr/Cu-Cu_2_O NPs film), (5% Cs/Agr/Cu-Cu_2_O NPs film) & (10% Cs/Agr/Cu-Cu_2_O NPs film)] were placed separately onto a 6 mm diameter well prepared by using a sterile cork borer on the previously inoculated agar plates applying Well Diffusion Method (Elboraey et al. 2021; Gamboa-Solana et al. 2021).These inoculated plates were incubated at 37 ℃ for 24 h. After incubation, the presence of a zone of inhibition was checked by visual inspection and the observed zones of inhibition (ZI) were measured in mm using a ruler and compared to the inhibition zone recorded by gentamicin 10 mcg (standard) susceptibility test discs (Sadia et al. 2021).

## Results and discussion

### Detection of ochratoxin a production by *A. niger* KY401431

**Fig. 1:**
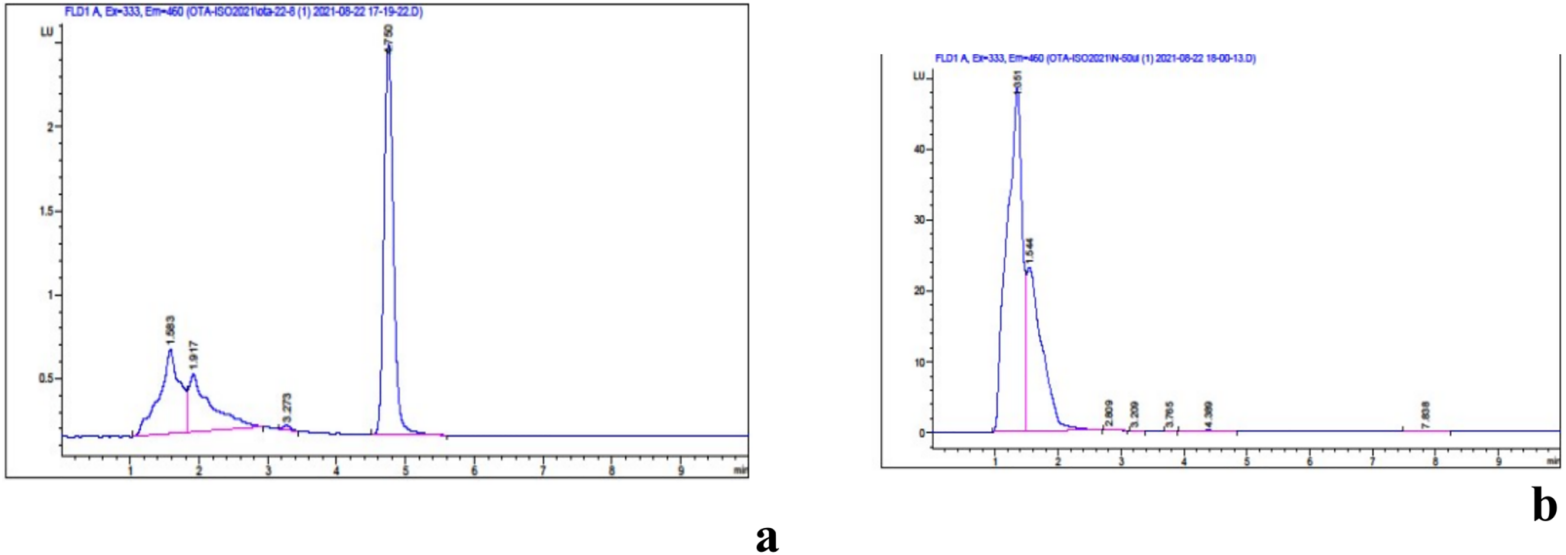
**a** HPLC chromatogram of Ochratoxin A standard **b**: HPLC chromatogram of *A. niger* K Y 401431 extract

Aspergillus niger K Y 401431 was checked for the production of the nephrotoxic and carcinogenic mycotoxin ‘ochratoxin A’ It is recommended to use strains of *A. niger* with inactive gene clusters for ochratoxins to prove the safety of the used strain prior to being accepted as a production strain. As shown in Fig. 1a OTA standard was being detected at 4.75 min. The absence of a corresponding peak in the extract obtained from *A. niger* Fig. 1b was observed.

### Optimization of the biosynthesized of Cu/Cu_2_O NPs using one-variable-at-a-time (OVAT) analysis

#### Effect of different concentration of copper sulphate (CuSO_4_) on the production of Cu/Cu_2_O NPs

**Fig. 2:**
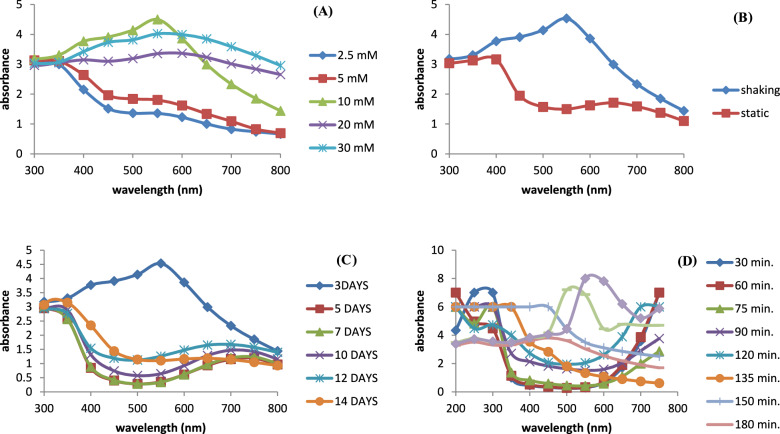
Effect of one variable at time in the synthesis of nanoparticles. **A** Effect of different concentration of copper sulphate (CuSO_4_) on the production of Cu/Cu_2_O NPs. **B** Effect of submerged and solid- state culture of *A. niger* on the production of Cu/Cu_2_O NPs. **C** Effect of different incubation periods of *A. niger* (culture age) on the production of Cu/Cu_2_O NPs under shaking culture conditions. **D** Effect of Reaction Time on the production of Cu/Cu_2_O NPs

From Fig. 2A, it is clear that formation of Cu/Cu_2_O NPs is markedly affected by copper sulphate concentration. It was observed that the highest absorption peak was recorded at a substrate concentration of 10 mM. Increasing the concentration of CuSO_4_ above 10 mM, the SPR bands became broad. On the other hand, lower concentrations of CuSO_4_ (2.5 and 5 mM) showed lower absorption peaks. Noor et al. (2020) reported that increasing salt concentration above 5 mM resulted in an increase of copper nanoparticle production. The highest peak was observed at 20 mM concentration.

#### Effect of submerged and solid-state culture of ***A. niger*** on the production of Cu/Cu_2_O NPs

Solid- state fermentation (SSF) presents advantages such as lower operating costs, lower space requirements, easier down-stream processing and simpler equipment (Li et al. 2021). In this study, biosynthesis of CuNPs using extracellular extract from solid-state fermentation and submerged fermentation (SmF) of *A. niger* was evaluated.

Figure 2B shows that the biosynthesized copper nanoparticles were formed using culture filtrate obtained either under static or shaking conditions. The copper SPR band of *Pseudomonas fluorescens* was reported to show an evident absorption peak between 550 and 650 nm. (Shantkriti and Rani 2014 and Bukhari et al. 2021) whereas, in another study the surface-plasmon resonance band of the copper or copper oxide nanoparticles was observed between 620 to 710 nm (Cuevas et al. 2015). However, using a culture filtrate developed under shaking conditions resulted in a sharper and higher absorbance peak at 550 nm. While, using culture filtrate obtained under static conditions resulted in a broad band shifted towards a less intense and longer wave length region (650 nm).

#### Effect of different incubation periods of ***A. niger*** (culture age) on the production of Cu/Cu_2_O NPs under shaking culture conditions

The UV–visible absorption spectroscopy of the biosynthesized Cu/Cu_2_O NPs was measured upon using different incubation periods of *A. niger* (3, 5, 7, 10, 12 and 14 days). As presented in Fig. 2C maximum absorbance was observed using the culture filtrate obtained after the incubation of *A. niger* for 3 days under shaking conditions. Older cultures showed a shift of the SPR band to 700 nm with lower peak intensity. Culture age affects Aspergillus niger's ability to synthesize CuO nanoparticles; metabolic changes and enzyme release during the stationary phase lead to an increase in nanoparticle formation. To have a better understanding of this process, more investigation into environmental variables and enzymatic pathways is required.

(Jain et al. 2015**)**.

In a study, for the effect of culture age on biosynthesis of gold nanoparticles using *A. Trinidadensis* (Deshmukh et al. 2023), it was found that 48 h culture age gave the highest yield, while older cultures resulted in lower yields. This was attributed to the possibility of releasing more enzymes required for gold reduction during log phase.

#### Effect of reaction time on the production of Cu/Cu_2_O NPs

In the present study, the effect of reaction time on the formation of copper nanoparticles was examined. The spectacular color change correlates with large shift of UV–Visible spectra. The first absorption peak of different curves is at 300 nm corresponding to oxidation product of culture filtrate. Figure 2D shows that at 135 min a second SPR band started to develop at 450 nm. The peak intensity of the developed SPR band increased in intensity at 150 min. At 180 min, the SPR band intensity (at 450 nm) decreased on increasing the reaction time. At 240 min, an SPR band at 550 nm characteristic for CuNPs is observed. This agrees with the time evolution of the dispersion photographs and the UV–Visible spectra found by Jain et al. 2015**.** It was found that increasing the reaction time to 6 h, the resulting copper nanoparticles displayed a broadened peak at 550 nm. Viet et al. (2016) reported the appearance of two peaks at 420 nm and 560 nm during preparation of CuNPs, the peak at 560 nm relates to existence of the CuNPs and the peak at 420 nm relates to the copper oxide nanoparticles.

### Characterization of biosynthesized Cu/Cu_2_O NPs produced by ***Aspergillus niger*** KY401431

#### Visual observation of biosynthesized Cu/Cu_2_O NPs

**Fig. 3:**
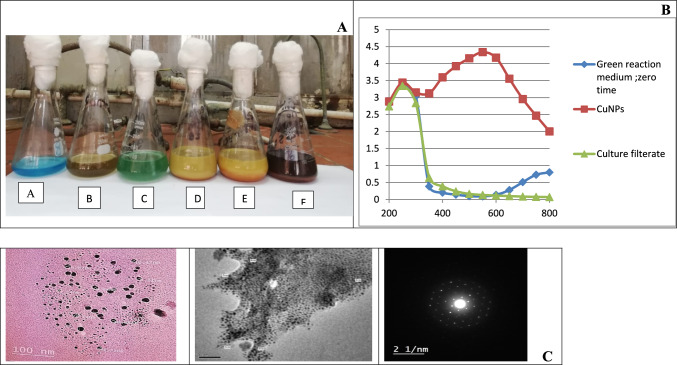
**A** Characterization of Cu/Cu_2_O NPs synthesis A: CuSO_4_ salt solution; B: culture filtrate; C: reaction at 0 time; D: reaction after 2 h; E: reaction after 3 h, F: reaction after 4 h. **B** UV–visible spectrum of culture filtrate of *A. niger* K Y 401431, green reaction medium at zero time and biosynthesized CuNPs. **C** HR- TEM image of biosynthesized Cu/Cu2O NPs

The first indication for Cu/Cu_2_O NPs synthesis in the aqueous solution is the colour change of the solution. For Cu/Cu_2_O NPs synthesis, the filtrate changed from yellow to brick red color indicating the formation of Cu/Cu2O NPs, while no color change was observed in the control as shown in Fig. 3A. This result agrees with Gaba et al. (2022) and Ingle et al. (2024)**.**

#### UV–visible spectroscopy

After 4 h, the green solution of the reaction medium turned into a dark reddish-brown colloidal suspension with λmax around 550 nm, confirming the synthesis of Cu/Cu_2_O NPs, as monitored by UV–Vis spectra in Fig. 3B. In the UV–Visible spectrum a strong broad peak was observed at 550 nm, and widening of the peak indicated that the particles were polydispersed. The SPR band's exact location may vary based on the size, shape of the nanoparticles and the metal precursor utilized and their concentration (Yang et al. 2016).

#### Transmission electron microscope (TEM) analysis

The TEM images of biosynthesized Cu/Cu_2_O NPs are presented in Fig. 3C. TEM images clearly show that Cu/Cu_2_O NPs were spherical shaped with diameter size of 10, 8 and 4 nm. Furthermore, the pictures clearly showed extracellular matrix and residual cellular debris. TEM analysis by Cuevas et al. (2015) confirmed the extracellular biosynthesis of spherical 5–20 nm copper and copper oxide nanoparticles synthesized by the fungus *Stereum hirsutum*.

#### Energy dispersive X-ray spectroscopy (EDX) analysis

**Fig. 4:**
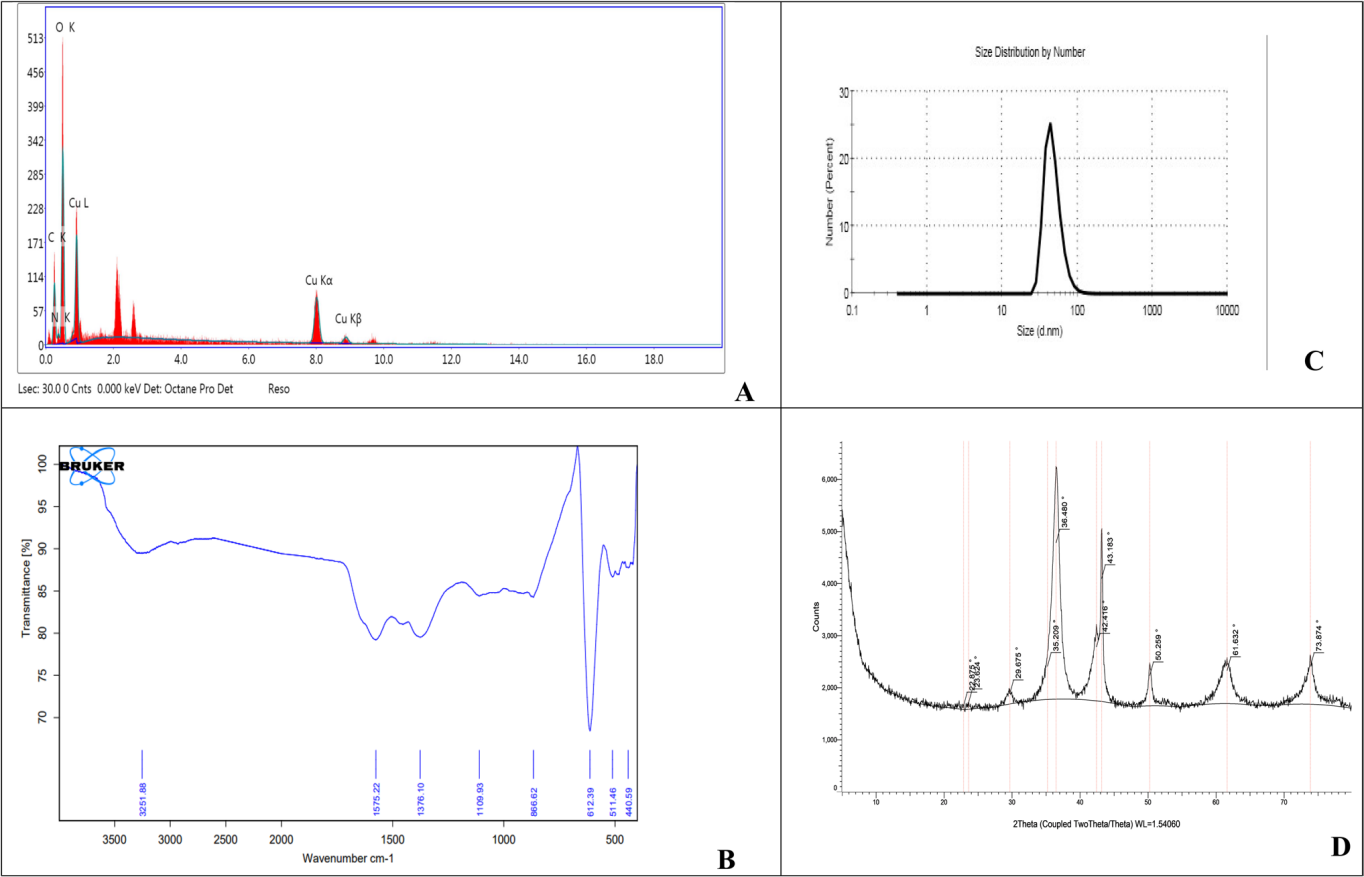
**A** EDX analysis of biosynthesized Cu/Cu_2_O NPs. **B** FTIR spectra of biosynthesized Cu/Cu_2_O NPs. **C** Particle size analysis of biosynthesized Cu/Cu_2_O NPs on the basis of DLS. **D** XRD of biosynthesized Cu/Cu_2_O NPs

The optical absorption peak from EDX spectroscopy was utilized to validate the existence of elemental Cu as shown in Fig. 4A. Results revealed the presence of Cu and O atoms that make up Cu/Cu_2_O NPs. Weaker peaks for C and N atoms are also detected due to the organic nature of the fungal extracellular extract used during the synthesis process. Bukhari et al. (2021) reported the EDX spectrum of copper oxide nanoparticles by Streptomyces sp. MHM38, showing a peak between 1, 8, and 9 keV.

#### Attenuated total reflection-Fourier transform infrared spectroscopy (ATR-FTIR) analysis

The ATR-FTIR spectra (Fig. 4B) of the biosynthesized Cu/Cu_2_O NPs showed bands in the region of 3251 cm^−1^, which is reported due to N–H moiety stretching vibrations of aliphatic primary amines overlapped with O–H stretching of hydroxyl groups. The carboxyl group's C–OH stretching vibration is represented by the peak at 1109 cm-1. The peak at 1575 correlates to the polysaccharide C = O moiety stretching or represent the protein amides I and II. The main stretch for copper nanoparticles was found at 612 cm^−1^ (Ullah et al. 2020; Nassar et al. 2023). FTIR analyses demonstrated that the proteins in the fungal extract cap Cu/Cu2O NPs in addition to reducing copper ions into copper nanoparticles. Thus, stabilizing the nanoparticles in aqueous medium.

#### Dynamic light scattering spectroscopy (DLS)

DLS of tested Cu/Cu_2_O NPs revealed a single peak with a polydispersed index (PdI) of 0.403 and an average hydrodynamic diameter size of 47.63 nm as shown in Fig. 4C. Regarding the sizes of the Cu/Cu_2_O NPs, it was found that those obtained by TEM differed considerably from the hydrodynamic diameters obtained with DLS.

Similar to our finding, a study conducted for the production of CuO NPs by using the fungal extract of *Ganoderma sessile*; Flores-Rabago (2023) reported the difference in sizes of CuO NPs by both analyses. It was suggested that this difference was due to the adhesion components (molecules and proteins) found in the extract which served as a capping agent to maintain the stability of NPs.

#### X-ray diffraction pattern of biosynthesized Cu/Cu_2_O NPs (XRD) analysis

An XRD analysis was performed (Fig. 4D), and the biosynthesized Cu/Cu_2_O NPs showed a powder X-ray diffractometry pattern with peaks as shown in Fig. 7 confirming the synthesis of CuNPs core covered with a shell of cuprous oxide (Cu_2_O), where the most distinctive diffraction peaks of elemental copper were found at 2ϴ = 43.183̊, 50.259̊, and 73.874̊ which represent the (1 1 1), (2 0 0) and (2 2 0) planes of copper's FCC structure and there are other peaks at 29.675̊, 36.480̊, 42.416̊ and 61.632̊ which describes the (1 1 0), (1 1 1), (2 0 0), and (2 2 0) plans of the Cu_2_O structure (Kaouti and Matouri 2010). Thus, the presence of Ců, Cu_2_O nanocrystals in the sample was confirmed, similarly Mohamed (2020) reported the presence of a Cu_2_O shell covering the copper core during the green synthesis of copper and copper oxide nanoparticles.

The Scherrer equation, which links the size of the crystalline domains to the broadening of the XRD peaks, can be used to estimate the average crystallite size of the nanoparticles. The study's comparatively narrow diffraction peaks indicate that the produced Cu/Cu2O nanoparticles have a small crystallite size, which may increase their surface area and reactivity and make them appropriate for a range of uses, including catalysts and antimicrobial agents (Kaouti and Matouri 2010).

### Characterization of Cs/Agr/Cu-Cu_2_O bionanocomposite film

#### SEM of bionanocomposite film

Because of its gelling qualities, biocompatibility, and capacity to create stable networks with polysaccharides like chitosan, agarose was selected as a stabilizer. Because of these properties, agarose works well to stabilize nanoparticles and stop them from aggregating. Its appropriateness for usage in bionanocomposites is further supported by the fact that agarose is widely employed in the pharmaceutical and biomedical industries (Wang et al. 2023**)**.

**Fig. 5:**
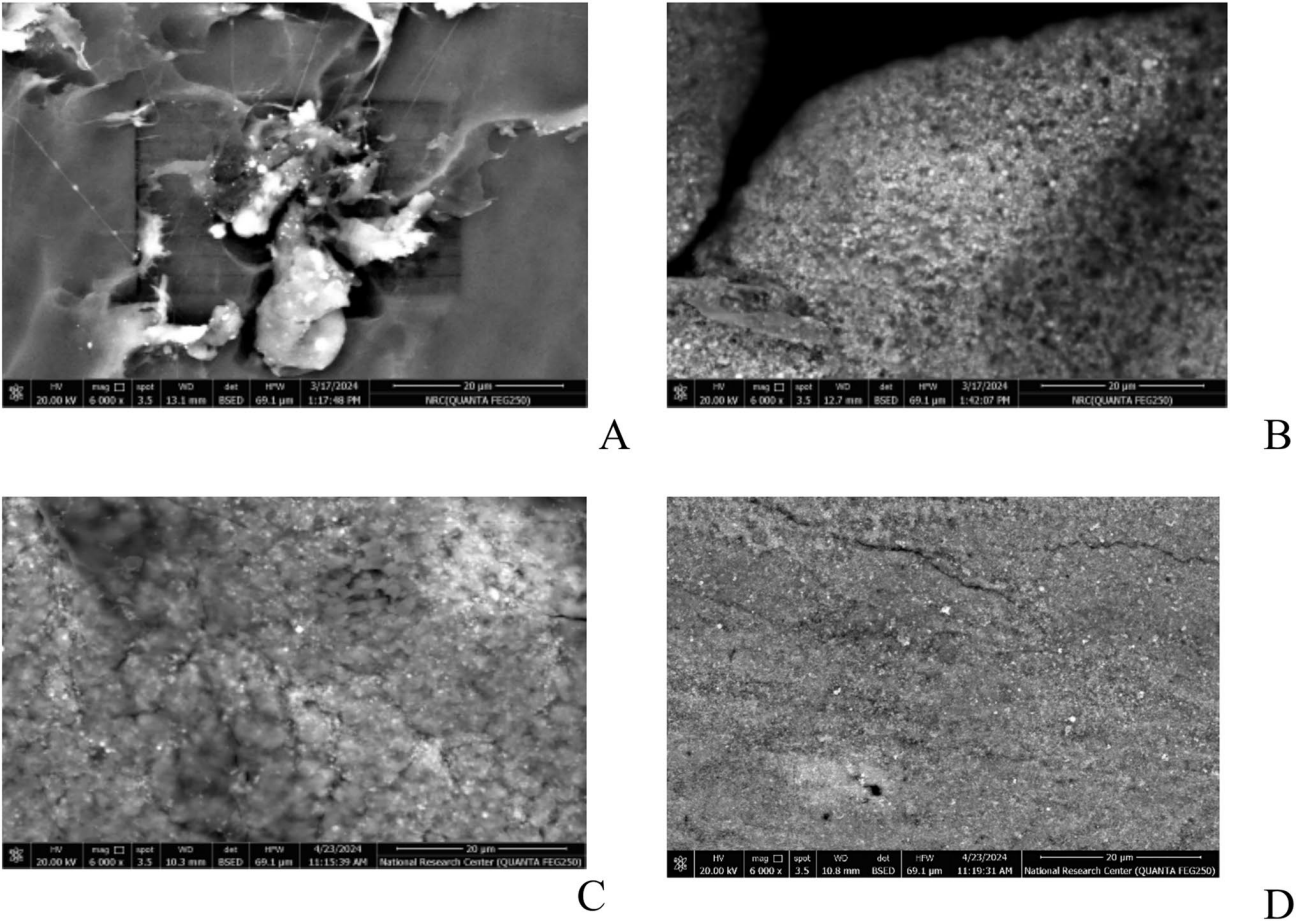
SEM images of **A** Cs/Agr blend and Cs/Agr/Cu-Cu_2_O bionanocomposite films containing different concentrations of Cu-Cu_2_O NPs, **B** 1%, **C** 5% and **D** 10%

The pure micrograph details of the pure chitosan-agarose composite film (Cs/Agr) and Cs/Agr/Cu-Cu_2_O bionanocomposite films containing different concentrations of the biosynthesized Cu-Cu_2_O NPs (1%, 5%, and 10%) were examined. SEM micrographs (Fig. 5) showed that there was a significant difference between the surface of the pure chitosan/agarose composite film before and after doping. Analysis shows that the surface of the pure chitosan/agarose is heterogeneous and after the addition of biosynthesized CuNPs the surface becomes homogenous. Cu-Cu_2_O NPs were uniformly distributed on the surface of Cs/Agr/Cu- Cu_2_O bionanocomposites matrix in case of addition of 1%, 5%, and 10% Cu-Cu_2_O NPs.

#### X-ray diffraction pattern of bionanocomposite film

**Fig. 6:**
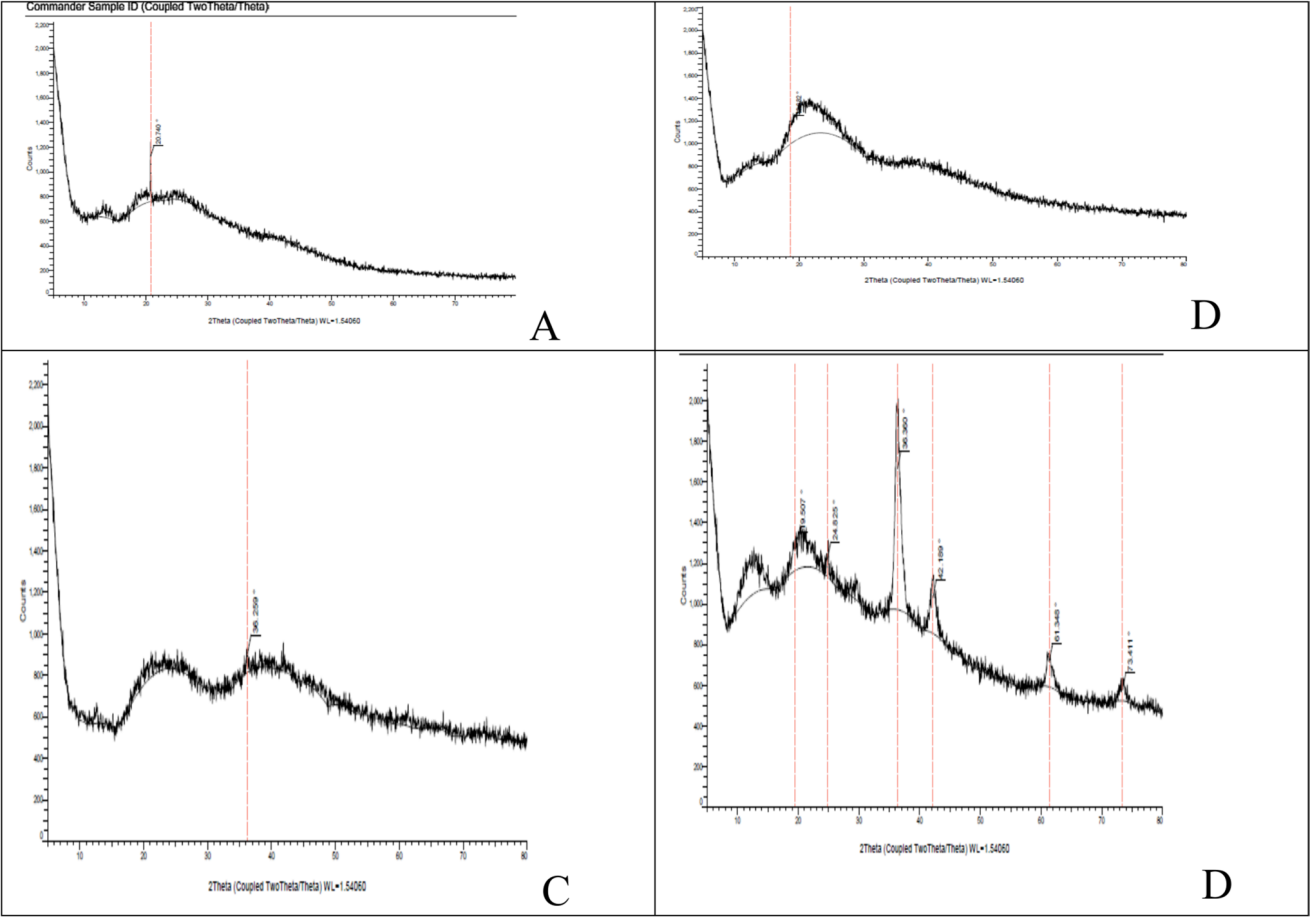
XRD patterns for: **A** chitosan/agarose and Cs/Agr/Cu-Cu_2_O bionanocomposite films loaded with **B** 1%, **C** 5% and **D** 10 wt% Cu-Cu_2_O NPs

The XRD patterns of Cs/Agr polymer blend film, and Cs/Agr/Cu-Cu_2_O bionanocomposite films are displayed in Fig. 6. The XRD profile of Cs/Agr blend (Fig. 6a) presented a sharp peak at 20.74̊ (2ϴ) expressing the fingerprint of the semicrystalline nature of chitosan (Govindan et al. 2012; Wang et al. 2023). The XRD analysis of Cs/Agr/Cu-Cu_2_O bionanocomposite films in case of 1% and 5% Cu-Cu_2_O NPs Fig. 6b, c displayed less defined peaks. The Cs/Agr/Cu-Cu_2_O bionanocomposite film at 10% Cu-Cu_2_O NPs (Fig. 6d) displayed characteristic peaks at 2ϴ = 19.5̊, 24.82̊, 36.56̊, 42.18̊, 61.34̊, and 73.41̊. This confirms the presence of chitosan and copper nanoparticles in the 10% bio-nanocomposite film. It is worth mentioning that all Cs/Agr/Cu-Cu2O bionanocomposite films (1%, 5%, and 10%) showed a considerable decrease in the intensity of the diffraction peak at 2ϴ = 20̊, and the peak widened, indicating the destruction of the chitosan's original crystalline structure. This implied that the introduction of copper–copper oxide nanoparticles reduced crystallinity and damaged symmetry and stereo-regularity by weakening the intra- and intermolecular hydrogen bonds in chitosan. Similar observation was mentioned by Wang et al. (2023).

#### Attenuated total reflection-Fourier transform infrared spectroscopy (ATR-FTIR) analysis of bionanocomposite films

**Fig. 7:**
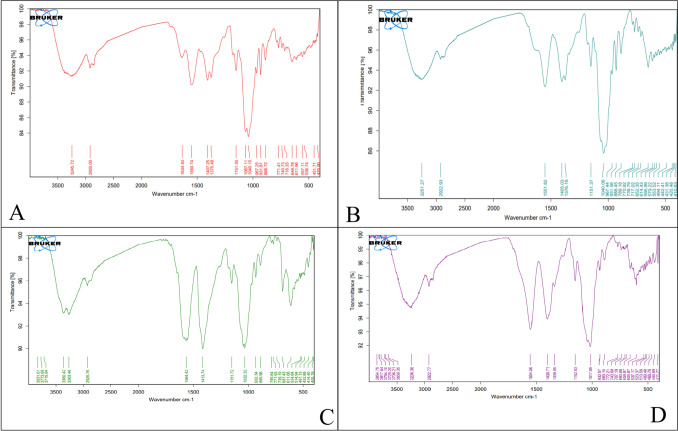
FTIR spectra for **A** chitosan agarose and Cs/Agr/Cu-Cu_2_O bionanocomposite films loaded with **B** 1%, **C** 5% and **D** 10 wt% Cu-Cu_2_O NPs

FT-IR spectroscopy measurements were performed to show possible intermolecular interactions among the system's components (Fig. 7). The spectrum of Cs/Agr (Fig. 7a) shows a strongly broad band between 3000 cm^−1^ and 3500 cm^−1^ assigned to both the intramolecular hydrogen bonds and the stretching vibrations of O–H and N–H. The absorption band at 2920 cm^−1^ can be attributed to C-H symmetric stretching. These bands are characteristics typical of polysaccharide. The bands located at approximately 1634 cm-1 (C = O stretching of amide I) verified the existence of residual N-acetyl groups and demonstrate the existence of acetyl amino groups, or a partially deacetylated form of chitin and at 1375 cm^−1^ is related to (C-N stretching of amide I). The N–H bending of amide II is represented by the band found at 1550 cm-1. The presence of bands at around 1407 and 1375 cm^−1^ refers to the CH2 bending and CH3 asymmetrical distortions. The band at 1067 cm^−1^ corresponds to the C-O stretching vibrations. The spectra of the chitosan samples published by Queiroz et al. (2014) include all of the previously mentioned bands. The absorption band at 1634 cm-1 is attributed to the O–H bending vibrations of agarose, whereas the distinctive absorption bands of 3, 6-anhydrogalactose and the C-H bending vibrations of anomeric carbon occurred at 931 cm-1 and 888 cm-1, respectively (Hu et al. 2012, 2016). A similar trend was observed in the Cs/Agr/Cu-Cu_2_O bionanocomposite films (Fig. 7 b, c, d) with slight variations. Disappearance of peak 1634 cm^−1^ in Fig. 7b, c, d indicates its involvement in complexation **(**Kaur et al. 2013). The band between 3300 cm^−1^ and 3500 cm^−1^ changed into sharper and stronger signal for Cs/Agr/Cu-Cu_2_O bionanocomposite films at 5% and 10% Cu-Cu_2_O NPs than that recorded for chitosan-agarose blend and 1% Cs/Agr/Cu-Cu_2_O bionanocomposite film. Similar observation was deduced for the peaks detected at 1554 and 1400 cm^−1^. The peak at 614 cm^−1^, 611 cm^−1^, 605 cm^−1^detected at 1%, 5%, and 10% Cu-Cu_2_O,respectively of the prepared Cs/Agr/Cu-Cu_2_O bionanocomposite films evidence the interaction between biosynthesized CuNPs and chitosan agarose composite (Usman et al. 2012; Manikandan and Sathiyabama 2015).

### Biological activities

#### [2, 2-Diphenyl-1-picrylhydrazil (DPPH) radical scavenging assay] of biosynthesized Cu/Cu_2_O NPs

The antioxidant activity of the biosynthesized Cu/Cu_2_O NPs was performed using DPPH method. The results confirmed that the biosynthesized Cu/Cu_2_O NPs has an antioxidant activity (85.2% ± 1.12) as compared to standard ascorbic acid (97.5% ± 0.74). In a study conducted by Ghareib et al. (2018), the DPPH scavenging assay was employed to explore the radical scavenging activity of the biosynthesized CuO NPs'by A. fumigatus in vitro.

At 100 µg/ml, the maximal scavenging activity of CuO NPs against DPPH was found to be 73.65%, compared to 88.13% for the standard antioxidant ascorbic acid.

#### In vitro cytotoxicity of biosynthesized Cu/Cu_2_O NPs

The exploration of nanomaterial-based cancer treatments, particularly those developed through environmentally friendly methods is extensive, with a focus on their potential integration into cancer therapy or their use as carriers or delivery systems for pharmaceuticals. The cytotoxicity of CuO NPs biosynthesized from various sources has been reported in several cancer cell lines (Zughaibi et al. 2022). In the present study the activity of biosynthesized Cu/Cu_2_O NPs against three cancer cell lines was investigated using the MTT assay method. The cytotoxic effect of the biosynthesized Cu/Cu_2_O NPs to lung, liver carcinoma human cell lines and Human Caucasian breast adenocarcinoma cell line namely, A549, HEPG-2, and MCF-7 showed IC_50_ of 20.3, 56.9, and 43.3 µg/ml, respectively. CuNPs'cytotoxic effects on cancer cells have been the subject of numerous reports. Fungal-mediated CuO-NPs were found to target MCF7 cancer cells at an IC50 concentration of 159.2 ± 4.5 µg/ml, according to Nassar et al. (2023). Liu et al. (2021) reported a reduction in HEPG2 cell viability with an IC50 value of 75 µg/ml. The therapeutic potential of nanoparticles is determined by their physical characteristics, including size, surface charge, and functional groups (Khan et al. 2019). Wongrakpanich et al. (2016) used the A549 human lung cell line to investigate the in vitro cytoxicity of CuO NPs of two different diameters (4 and 24 nm). 24 nm CuO NPs were noticeably more cytotoxic than 4 nm CuO NPs, even though they had same surface and core oxide structure. On the contrary, using the MTT technique, Abbasi et al. (2021) found that 30 nm nanoparticles had greater anti-cancer potential than 60 nm CuO NPs.

Despite having promising antibacterial qualities, films based on Cu/Cu_2_O nanoparticles raise a number of safety issues that should be resolved before being used in human applications. According to cytotoxicity studies, Cu and Cu2O nanoparticles can be harmful, especially when present in high concentrations. Oxidative stress and DNA damage are two such hazards. To assess these films'biocompatibility in healthy human tissues, more research on non-cancerous cell lines is required (Khan et al. 2019).

#### Antibacterial activity of Cs/Agr/Cu-Cu_2_O bionanocomposite films

**Fig. 8:**
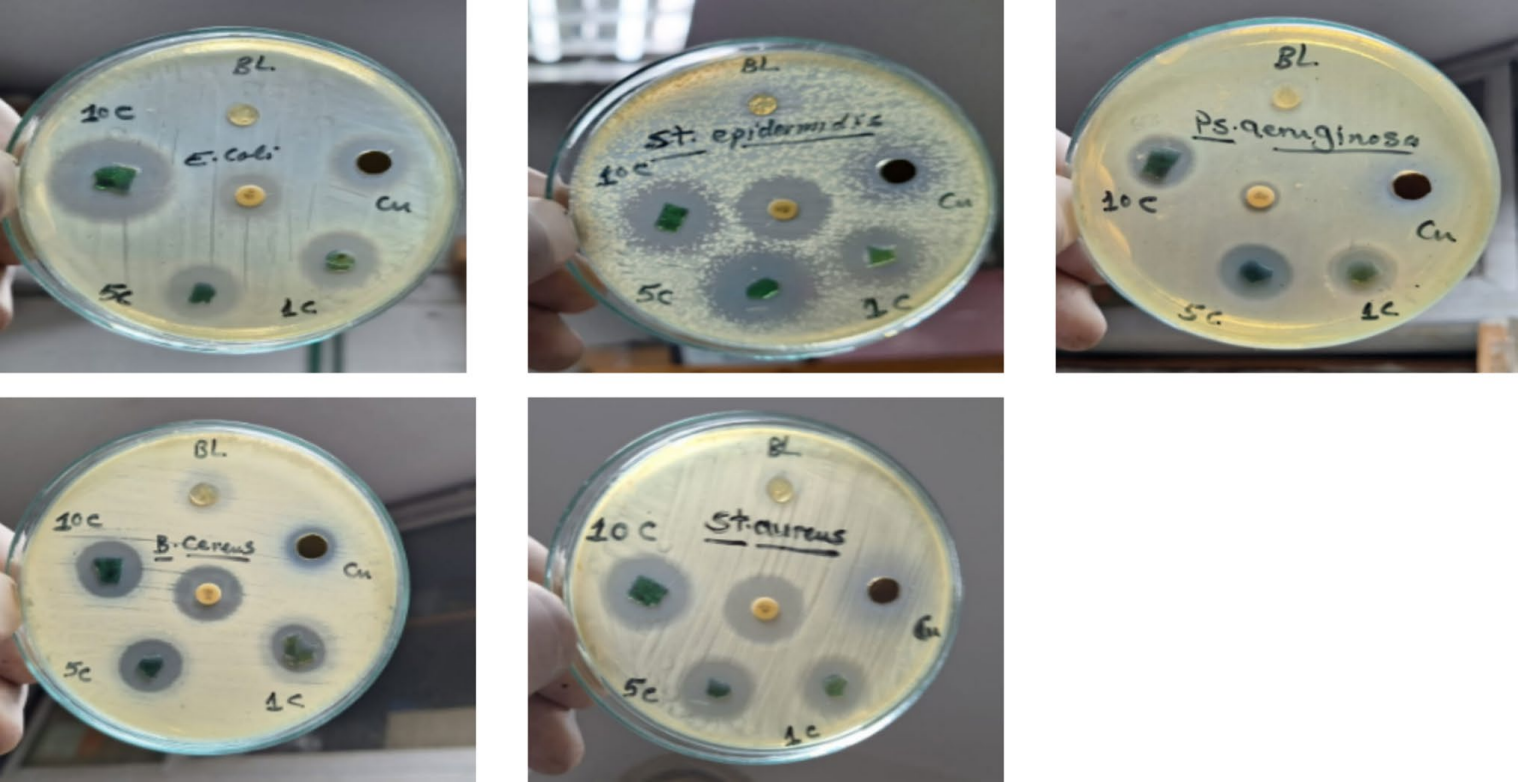
Antibacterial activity of biosynthesized Cu/Cu_2_O NPs and chitosan agarose nanocomposite films loaded with different concentrations of biosynthesized Cu/Cu_2_O NPs. BL: Cs/Agr composite, Cu: biosynthesized Cu/Cu_2_O NPs and Cs/Agr/Cu- Cu_2_O bionanocomposite films loaded with; 1c: 1%, 5c: 5% and 10c: 10% wt% Cu- Cu_2_O NPs

The disc diffusion method was utilized to determine the pathogenic strains'susceptibility. The inhibitory activity was measured based on the clear zone of inhibition. The observed zone of inhibition clearly demonstrates that Cu/Cu2O nanoparticles exhibit significant bactericidal efficacy (Fig. 8). The ability of Cs/Agr/Cu-Cu_2_O bionanocomposite films to prevent the growth of the examined pathogenic strains is also shown in Fig. 8. Our results (Table 1) revealed that the chitosan agarose stabilized Cu-Cu_2_O NPs demonstrated boosted inhibitory efficacy against both gram-negative and gram-positive bacteria than the individual biosynthesized Cu-Cu_2_O NPs and the chitosan agarose composite film. It was also shown that the diameter of the inhibition zone increased with increasing Cu-Cu2O NP loading in the prepared Cs/Agr/Cu-Cu_2_O bionanocomposite films. At a concentration of 10% Cu-Cu_2_O NPs for preparation of Cs/Agr/Cu-Cu_2_O NPs bionanocomposite film; the inhibition zone recorded exceeded that attained by the standard Gentamicin antibiotic against *Escherichia coli* ATCC 25922, *Pseudomonas aeruginosa* ATCC 27853, *Bacillus cereus* ATCC 6629 and *Staphylococcus epidermidis* ATCC 12228. As CuNps adhere to a bacterium's surface, it changes the characteristics of its membrane, which causes cell death. (Li et al. 2008). Chitosan agarose composite demonstrated only zone of inhibition against Bacillus cereus and Staphylococcus epidermidis. In a comparative study (Manikandan and Sathiyabama 2015), the negligible zone of inhibition demonstrated by chitosan has been attributed to the absence of chitosan's antibacterial action at p H 7.4; the Muller-Hinton agar's p H, whereas in our study; nutrient agar medium (NA) of p H 6.8 was used. This supports the idea that chitosan only exhibits antibacterial activity in acidic media because it is poorly soluble above pH 6.5 (Rabea et al. 2003).

**Table 1:** Inhibition zone diameter (millimeter) of the samples applying well diffusion method

Tested bacteria	Inhibition zone mm					
	blank	CuNPs	1%CS/Agr/Cu- Cu_2_O NPs	5% CS/Agr/Cu- Cu_2_O NPs	10% CS/Agr/Cu- Cu_2_O NPs	Gentamicin
*Escherichia coli* ATCC 25922	–	15	17	19	25	19
*Pseudomonas aeruginosa* ATCC 27853	–	12	13	17	17	10
*Bacillus cereus* ATCC 6629	13	13	17	18	20	18
*Staphylococcus aureus* ATCC 6538	–	12	12	16	18	20
*Staphylococcus epidermidis* ATCC 12228	8	13	16	21	21	15

*GN* GENTAMICIN 10 mcg (standard antibiotic disc)

*ND* Not detected

*Blank* Chitosan agarose composite film

## Conclusion

In the present study, *Aspergillus niger* K Y 401431 cell-free culture filtrate was used for biosynthesis of copper/copper oxide nanoparticles in an eco-friendly process. The influence of some parameters was investigated for the optimized production of copper/copper oxide nanoparticles. The characterization of Cu/Cu_2_O NPs revealed the formation of crystalline spherical shaped (Cu/Cu_2_O NPs) with 4–10 nm size. Anticancer activity of the biosynthesized Cu/Cu_2_O NPs was studied. MTT assay showed that biosynthesized Cu/Cu_2_O NPs have a remarkable cytotoxic effect against three cancer cell lines MCF7, HEPG2 and A549. The biosynthesized Cu/Cu_2_O NPs were used for the preparation of bionanocomposite films with the addition of chitosan and agarose. The Cs/Agr/Cu-Cu_2_O bionanocomposite film displayed enhanced antibacterial activity against pathogenic bacteria. This new Cs/Agr/Cu- Cu_2_O bionanocomposite film can be employed as a sustainable and environmentally friendly food packaging material and for biomedical applications (e.g., wound dressings).
